# Supplementary material for: The Small RNA Universe of Capitella teleta
Source: Front Mol Biosci. 2022 Feb 25;9:802814. doi: 10.3389/fmolb.2022.802814 (PMC8915122; doi:10.3389/fmolb.2022.802814)
Supplement: Supplementary file 1 [file DataSheet1.ZIP › Supplement/known/CAPTEscaffold_933_30851.pdf]

Provisional ID	:	CAPTEscaffold_933_30851
Score total	:	15839.8
Score for star read(s)	:	3.9
Score for read counts	:	15829.7
Score for mfe	:	1.6
Score for randfold	:	1.6
Score for cons. seed	:	3
Total read count	:	31061
Mature read count	:	29933
Loop read count	:	0
Star read count	:	1128

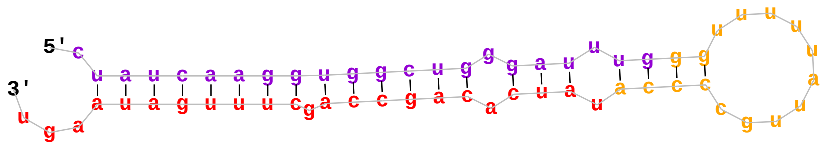

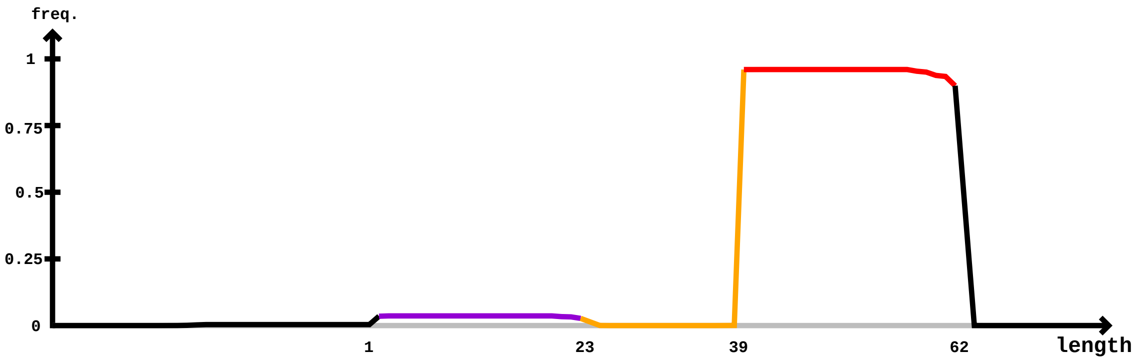


```
                                   Star                                  Mature
5'- guuuuuguuuguguuuuuggguuugaucggccuaucaagguggcugggauuuggguuuuuauugccccauaucacagccagcuuugauaaguugacugaccuucagu   -3'   obs
    guuuuuguuuguguuuuuggguuugaucggccuaucaagguggcugggauuuggguuuuuauugccccauaucacagccagcuuugauaaguugacugaccuucagu         exp
    guuuuuguuuguguuuuuggguuugaucggccuaucaagguggcugggauuuggguuuuuauugccccauaucacagccagcuuugauaaguugacugaccuucagu         known
    ..................(((((.(.(((((.((((((((((((((.(((.((((..........)))).))).)))))).)))))))).)))))).))))).....    reads   mm   sample
    ...uuuguuuguguuuuuggguuugaucggc............................................................................    1       0    seq
    .........uguguuuuugggguCugaucggc...........................................................................    1       1    seq
    ..........guguuuuuggguuugaucggc............................................................................    4       0    seq
    ...........uguuuuuggguuugaucggc............................................................................    23      0    seq
    ............guuuuuggguuugaAcggc............................................................................    1       1    seq
    ............guuuuugAguuugaucggc............................................................................    1       1    seq
    ............guuuuuggguuugaucggc............................................................................    48      0    seq
    .............uuuuuggguuugaucggc............................................................................    35      0    seq
    .............uuuuugggCuugaucggc............................................................................    1       1    seq
    ...............................cuGucaagguggcugggau.........................................................    1       1    seq
    ...............................cuaucaagguggcugggau.........................................................    83      0    seq
    ...............................cuaucaagguAgcugggau.........................................................    1       1    seq
    ...............................cuaucaagguggcugggauu........................................................    24      0    seq
    ...............................cuaucaagguggcugggauA........................................................    4       1    seq
    ...............................cuaucaagguggcuAggauuu.......................................................    1       1    seq
    ...............................cuaucaagguggcugggauuu.......................................................    172     0    seq
    ...............................Nuaucaagguggcugggauuu.......................................................    1       1    seq
    ...............................cuaucaagguggcugAgauuug......................................................    1       1    seq
    ...............................cuaucaGgguggcugggauuug......................................................    3       1    seq
    ...............................cuaucaagguggcuAggauuug......................................................    1       1    seq
    ...............................cuaucaagguggcugggauuAg......................................................    1       1    seq
    ...............................cuaAcaagguggcugggauuug......................................................    1       1    seq
    ...............................cuaucaagguggcugggauuuA......................................................    1       1    seq
    ...............................cuaucaagguggcugggauuug......................................................    749     0    seq
    ...............................cuGucaagguggcugggauuug......................................................    3       1    seq
    ...............................cuaucaagguggcugggauuugA.....................................................    10      1    seq
    ...............................cuaucaagguggcugggauuugg.....................................................    3       0    seq
    ...............................cuaucaagguggcugggauuugU.....................................................    25      1    seq
    ...............................cuaucaagguggcugggauuuggU....................................................    1       1    seq
    ...............................cuaucaagguggcugggauuuggg....................................................    4       0    seq
    ................................uaucaagguggcugggauu........................................................    1       0    seq
    ................................uaucaagguggcugggauuug......................................................    5       0    seq
```

Star **Mature**

guuuuuuguuuguguuuuuggguuugaucggccuaucaagguggcugggauuugggguuuuuauugccccauaucacagccagcuuugauaaguugacugaccuucagu			
.................................uaucaagguggcugggauuugg...	29	0	seq
.................................uaucaagguggcugggauuuggA..	1	1	seq
.................................uaucaagguggcugggauuuggU..	2	1	seq
....cauaucacagccagcuuuga....................	3	0	seq
....cauaucacagccagcuuugau...................	1	0	seq
....cauaucacagccagcuuugaua..................	1	0	seq
....cauaucacagccagcuuugauaa.................	1	0	seq
....auaucacagccagcuuugau...................	4	0	seq
....auaucacagccagcuuugauaag................	1	0	seq
....auaucacagccagcuuugauaagu...............	2	0	seq
....uaucacagccagcuuuga....................	192	0	seq
....uaucacagUcagcuuuga....................	1	1	seq
....uaucacagccagcuGuga....................	1	1	seq
....uaucacagccagcuuAga....................	1	1	seq
....uaucacagccagcuuugaA...................	3	1	seq
....uaucacagccagcuuugau...................	97	0	seq
....uaucacagccagcuuugaAa..................	2	1	seq
....uaucacagccagcAuugaua..................	1	1	seq
....Aaucacagccagcuuugaua..................	1	1	seq
....uaucacagccagcuuugaua..................	374	0	seq
....uaucacagccagcuuugauU..................	5	1	seq
....uaucacagccagcuuAgaua..................	1	1	seq
....uaucacGgccagcuuugaua..................	2	1	seq
....uaucacagccagcuuuUaua..................	1	1	seq
....uaucacagccagcuuAgauaa.................	1	1	seq
....uaucacagAcagcuuugauaa.................	1	1	seq
....uaucacaCccagcuuugauaa.................	1	1	seq
....uaucacagccagcuuugauaa.................	118	0	seq
....uaucacagccagcuuugauaag................	1062	0	seq
....uaucacagccagcuuugaCaag................	1	1	seq
....uaucacagccagcuuugauaaA................	5	1	seq
....uaAcacagccagcuuugauaag................	1	1	seq
....uaucacagAcagcuuugauaag................	1	1	seq
....uaucacagccagcuuAgauaag................	1	1	seq
....uaucUcagccagcuuugauaag................	1	1	seq
....uaucacagccagcuAugauaag................	1	1	seq
....uaucacagcAagcuuugauaag................	3	1	seq
....Aaucacagccagcuuugauaag................	4	1	seq
....uauUacagccagcuuugauaag................	1	1	seq
....uaucacagccagcuuugaAaag................	1	1	seq
....uaucacagccagcuCugauaag................	1	1	seq
....uaucacGgccagcuuugauaag................	3	1	seq
....uauGacagccagcuuugauaagu...............	1	1	seq
....uaucacagccagcGuugauaagu...............	2	1	seq
....uaucacagccaCcuuugauaagu...............	1	1	seq
....uaucacagccagcuGugauaagu...............	1	1	seq
....uaucacagccagcuuugauaagG...............	16	1	seq
....uaucacaAccagcuuugauaagu...............	6	1	seq
....uaucacagccagcuuugauaagA...............	28	1	seq
....uauAacagccagcuuugauaagu...............	6	1	seq
....uaucacagccagcCuugauaagu...............	16	1	seq
....uaucaGagccagcuuugauaagu...............	2	1	seq
....uaucacGgccagcuuugauaagu...............	24	1	seq
....uaucacagccagAuuugauaagu...............	12	1	seq
....uaucacagccagcuAugauaagu...............	8	1	seq
....uaucacagccagcuuugauCagu...............	1	1	seq
....uaucacagccagcuuugauaagC...............	2	1	seq
....Naucacagccagcuuugauaagu...............	6	1	seq
....uaAcacagccagcuuugauaagu...............	34	1	seq
....uaucacagccagcuuugauGagu...............	25	1	seq
....uaucacagccagcuCugauaagu...............	2	1	seq
....uaucacagccagcuuugaGaagu...............	2	1	seq
....uaucacagcAagcuuugauaagu...............	13	1	seq
....uaucacagcUagcuuugauaagu...............	10	1	seq
....uaucacagccagUuuugauaagu...............	1	1	seq
....uaucacagccagcuuugUuaagu...............	5	1	seq
....uaucacagccagcuuugauaaCu...............	3	1	seq
....uaucacagccagcuuugauaaAu...............	2	1	seq
....uaucacagccagcuuuAauaagu...............	21	1	seq
....uGucacagccagcuuugauaagu...............	5	1	seq

Star Mature

guuuuuuguuuguguuuuuggguuugaucggccuaucaagguggcugggauuuggguuuuuauugccccauaucacagccagcuuugauaaguugacugaccuucagu			
....uaucacagccagcuuugauaagu...............	27358	0	seq
....uaucacagccagcuuugauaUgu...............	2	1	seq
....uaCcacagccagcuuugauaagu...............	3	1	seq
....Aaucacagccagcuuugauaagu...............	100	1	seq
....uaucUcagccagcuuugauaagu...............	2	1	seq
....uaucacagUcagcuuugauaagu...............	2	1	seq
....uaucacagccagcuuCgauaagu...............	6	1	seq
....uUucacagccagcuuugauaagu...............	3	1	seq
....uaucGcagccagcuuugauaagu...............	4	1	seq
....uaucCcagccagcuuugauaagu...............	1	1	seq
....uaucacagccagcuuugGuaagu...............	4	1	seq
....uaucacagccCgcuuugauaagu...............	1	1	seq
....Caucacagccagcuuugauaagu...............	6	1	seq
....uaucacagccagcAuugauaagu...............	3	1	seq
....uaucaAagccagcuuugauaagu...............	8	1	seq
....uaucacaUccagcuuugauaagu...............	5	1	seq
....uaucacagAcagcuuugauaagu...............	10	1	seq
....uaucacagccagcuuuUauaagu...............	2	1	seq
....uaucacagccagGuuugauaagu...............	1	1	seq
....uNucacagccagcuuugauaagu...............	1	1	seq
....uauUacagccagcuuugauaagu...............	16	1	seq
....uaucacaCccagcuuugauaagu...............	3	1	seq
....Gaucacagccagcuuugauaagu...............	5	1	seq
....uaucacagccagcuuugauaGgu...............	4	1	seq
....uaucacagccagcuuugauaCgu...............	1	1	seq
....uaucacagccGgcuuugauaagu...............	2	1	seq
....uaucacagcGagcuuugauaagu...............	3	1	seq
....uaucacagccaAcuuugauaagu...............	2	1	seq
....uaucacagccagcuuugaAaagu...............	7	1	seq
....uaucacagccagcuuAgauaagu...............	20	1	seq
....uaucaNagccagcuuugauaagu...............	1	1	seq
....uaucacagccagcuuuCauaagu...............	3	1	seq
....uaucacagccagcuuugaCaagu...............	5	1	seq
....uaucaUagccagcuuugauaagu...............	5	1	seq
....uaucacagccUgcuuugauaagu...............	2	1	seq
....uaucacUgccagcuuugauaagu...............	5	1	seq
....uaucacagAcagcuuugauaaguu..............	1	1	seq
....uaucacagccagcuuugauaaguu..............	106	0	seq
....uaucacagccagcuuugauaaguA..............	54	1	seq
....uaAcacagccagcuuugauaaguu..............	1	1	seq
....uaucacagccagcuuCgauaaguu..............	1	1	seq
....uaucacagccagcuuugauaaguuU.............	4	1	seq
....ucacagccagcuuugauaagu...............	1	0	seq
....cacagccagcuuugauaag................	1	0	seq
....acagccagcuuugauaagu...............	1	0	seq
